# Biarsenical ligands bind to endogenous G-protein α-subunits and enable allosteric sensing of nucleotide binding

**DOI:** 10.1186/1471-2091-14-37

**Published:** 2013-12-17

**Authors:** Lauri Tõntson, Sergei Kopanchuk, Ago Rinken

**Affiliations:** 1 University of Tartu, Institute of Chemistry, Ravila 14a, 50411, Tartu, Estonia; 2 Competence Centre on Reproductive Medicine & Biology, Tiigi 61b, 50410, Tartu, Estonia

**Keywords:** G-proteins, Tetracysteine, F_2_FlAsH, Fluorescence anisotropy, Nucleotide binding

## Abstract

**Background:**

Heterotrimeric G-proteins relay extracellular signals to intracellular effector proteins. Multiple methods have been developed to monitor their activity; including labeled nucleotides and biosensors based on genetically engineered G-proteins. Here we describe a method for monitoring unlabeled nucleotide binding to endogenous G-proteins α-subunits in a homogeneous assay based on the interaction of 4′,5′-bis(1,2,3-dithioarsolan-2-yl)-2′,7′-difluorofluorescein (F_2_FlAsH) with G-protein α-subunits.

**Results:**

The biarsenic fluorescent ligand F_2_FlAsH binds to various wild-type G-protein α-subunits (αi1, αi2, αi3, αs_long_, αs_short_, αolf, αq, α13) via high affinity As-cysteine interactions. This allosteric label enables real time monitoring of the nucleotide bound states of α-subunits via changes in fluorescence anisotropy and intensity of their F_2_FlAsH-complexes. We have found that different α-subunits displayed different signal amplitudes when interacting with F_2_FlAsH, being more sensitive to nucleotide binding to αi, αs, αolf and αq than to α13. Addition of nucleotides to F_2_FlAsH-labeled α-subunits caused concentration-dependent effects on their fluorescence anisotropy. pEC_50_ values of studied nucleotides depended on the subtype of the α-subunit and were from 5.7 to 8.2 for GTPγS, from 5.4 to 8.1 for GppNHp and from 4.8 to 8.2 for GDP and lastly up to 5.9 for GMP. While GDP and GMP increased the fluorescence anisotropy of F_2_FlAsH complexes with αi-subunits, they had the opposite effect on the other αβγM complexes studied.

**Conclusions:**

Biarsenical ligands interact allosterically with endogenous G-protein α-subunits in a nucleotide-sensitive manner, so the presence or absence of guanine nucleotides has an effect on the fluorescence anisotropy, intensity and lifetime of F_2_FlAsH-G-protein complexes.

## Background

Heterotrimeric guanine nucleotide binding proteins (G-proteins) play an integral role in signal transduction and, when activated by G-protein coupled receptors (GPCRs), relay signals crossing the plasma membrane to intracellular effector proteins. They are composed of α- and βγ-subunits and bind guanosine diphosphate (GDP) in their inactive and guanosine triphosphate (GTP) in their activated state. In the latter case the heterotrimer may dissociate fully or partially and both subunits may subsequently interact with downstream effectors [1]. Intrinsic GTPase activity of the α-subunit leads to eventual inactivation, completing the cycle as the subunits reassociate. Various means have been employed to measure the activity of G-proteins, with measurements of GTPase activity, intrinsic fluorescence and binding of radioactively or fluorescently labeled nucleotides being among the most common methods [2]. Genetic engineering has made possible the development of G-proteins that are either fused to fluorescent proteins or contain a motif that allows for their labeling with various small fluorescent molecules [3]. One such motif is the tetracysteine tag, which binds 4′,5′-bis(1,3,2-dithioarsolan-2-yl)fluorescein (FlAsH) analogues with high affinity and selectivity [4]. It has been used to label G-proteins to give resonance energy transfer pairs with either another also fluorescently labeled G-protein subunit [5,6], or other proteins that interact with G-proteins such as GPCRs or regulators of G-protein signaling [2,7]. One disadvantage of this method is that the interacting proteins have to be labeled with donor and acceptor fluorophores, which limits the range of interactions that can be measured and can lead to alterations in their function, while simultaneously conferring selectivity due to the requirement for close proximity between the donor and acceptor for efficient energy transfer.

Fluoresceine-based biarsenical fluorophores such as FlAsH or F_2_FlAsH retain the parent compounds hydrophobicity and may bind nonspecifically to intracellular proteins and membranes [4], which can generate high background signals. This kind of interaction can become advantageous when FlAsH-analogues are used as sensitive reporters of their molecular environment: for example as conformation sensitive probes of protein structure [8]. FlAsH is also known to bind to cysteine-rich proteins *in vivo*[9]. Taking advantage of these properties, we have developed a method for *in vitro* monitoring of nucleotide binding to heterotrimeric G-proteins based on F_2_FlAsH interactions with cysteine residues of endogenous G-protein α-subunits. We have used this method to characterize nucleotide binding to 8 different G-proteins and show that F_2_FlAsH interactions with G-proteins are subtype specific.

## Methods

### Cell lines and reagents

Spodoptera frugiperda 9 (Sf9) cells were from Invitrogen Life Technologies (Carlsbad, CA, USA). HEPES, NaCl, EDTA, MgCl_2_ were from Applichem GmbH (Darmstadt, Germany). GDP, guanosine monophosphate (GMP), guanosine 5′–O-[gamma-thio]triphosphate (GTPγS), guanosine 5′-[β,γ-imido]triphosphate (GppNHp), dodecylsucrose, sodium cholate, polyoxyethylene (10) lauryl ether (C12E10), tris(2-carboxyethyl)phosphine (TCEP), ethanedithiol, desthiobiotin were from Sigma-Aldrich GbmH (Munich, Germany). AsCl_3_ was from Reachim (Russia). β-mercaptoethanol was from Merck KGaA (Darmstadt, Germany). F_2_FlAsH was synthesized according to published procedures [10]. FlAsH was from Toronto Research Chemicals (Toronto, Canada). G-protein α-subunits (αq, αs_long_, αs_short_, αolf and α13) were from Kerafast Inc, (Boston, MA, USA). Tetracysteine-labeled peptide (FLNCCPGCCMEP) was from Bachem AG (Bubendorf, Switzerland). Pyruvate kinase was from Roche diagnostics GmbH (Mannheim, Germany), BSA was from PAA Laboratories GmbH (Pasching, Austria). Fluorescein was from Lambert Instruments (Roden, the Netherlands).

### Protein expression and purification

G-protein αi1, αi2, αi3 and dual-tagged β1γ2-subunits (βγM) were expressed and purified as previously described [11] using tandem affinity chromatography [12]. Briefly, Sf9 cells were grown in serum free medium in shaker flasks and infected with baculoviral stocks to simultaneously express either only βγM-subunits or βγM and αi-subunits. Infected cells were harvested after 48 h. Cell pellets were homogenized in ice cold homogenization buffer (HB: 20 mM HEPES, pH = 8, 10 mM NaCl, 2 mM MgCl_2_, 1 mM EDTA, 5 μM GDP, 5 mM β-mercaptoethanol and protease inhibitors, diluted according to manufacturer’s recommendations: Roche Complete EDTA-free, Roche diagnostics GmbH (Mannheim, Germany)). Cells were homogenized by sonication for 5 cycles of 10 sec (Bandelin SonoPuls, Bandelin electronic GmbH, Berlin, Germany). Homogenates were then centrifuged for 30 min at 40 000 × g (Sigma 3 K30, SIGMA Laborzentrifugen GmbH, Osterode am Harz, Germany) and the resulting membrane pellets resuspended in solubilization buffer (HB with 1% Na-cholate, 0,1% C12E10 and 0,5% dodecylsucrose) and shaken for 1 h at 4°C at 250 rpm (ELMI DOS-20S, ELMI Ltd, Riga, Latvia). The solubilized proteins were separated by centrifugation for 30 minutes at 40 000 × g and purified with affinity chromatography using Strep-Tactin Superflow high capacity resin (IBA GmbH, Göttingen, Germany) in Poly-Prep columns (Bio-Rad, Hercules, CA, USA). The columns were washed with washing buffer (WB: 20 mM HEPES, pH = 8, 10 mM NaCl, 1 mM EDTA, 0,5% C12E10, 5 mM β-mercaptoethanol) and the G-proteins eluted with elution buffer (WB +2 mM desthiobiotin). Eluates were aliquoted, frozen and kept until use at −80°C. Protein concentrations were determined by UV-absorbance at 280 nm using a Nanodrop 1000 spectrophotometer (NanoDrop products, Wilmington, DE, USA) and purities estimated using Ag-stained SDS-PAGE gels [11].

### Fluorescence lifetime measurements

We determined fluorescence lifetimes in the frequency domain using an imaging attachment (LIFA-X, Lambert Instruments, Roden, The Netherlands) consisting of a signal generator, Multi-LED excitation source with a 3 W light emitting diode (477 nm LED), and an intensified CCD Li^2^CAM-X with GEN-III GaAs photocathode. The CCD was mounted on the side port of an iMIC inverted digital fluorescence microscope (Till Photonics GmbH, Gräfelfing, Germany) through a TuCam adapter with 2× magnification (Andor Technology, Belfast, UK). Multi-LED was fiber coupled to the epicondenser of iMIC. The filter cube comprised of a BrightLine HC 475/35 nm (Semrock, New York, USA) exciter, a zt 491 rdcxt dichroic (Chroma, Bellows Falls, USA) and a BrightLine HC 525/45 nm (Semrock, New York, USA) emitter. For all samples and references a series of images with an exposure time of 150 ms was taken at 11 modulating frequencies (from 1–120 MHz, with increasing LED AC from 0.1 until 2.5 V) and 12 phase-shifts between LED and image intensifier per every modulating frequency. MCP gain used was 750. Photons were collected with 4 × UPLSAPO objective (Olympus, Japan). To increase efficiency of primary photon collection and to decrease the effect of photobleaching 4 images/phase were averaged. Camera binning of 4 by 4 was used. For lifetime calibration, a solution of fluorescein (0.1 μM, at pH > 10) was used as reference with τ = 4.02 ns. The background correction was performed automatically by subtracting an image obtained with blocked excitation using Li-FLIM v1.2.22 software (Lambert Instruments, Roden, The Netherlands).

Samples were measured in 4 well CMS Chamlide chambers (Live Cell Instrument, Seoul, Korea) with 1.5H cover glasses (Paul Marienfeld GmbH & Co, Lauda-Königshofen, Germany).

By using nonlinear optimization routines (Levenberg-Marquart) the distance between measured modulation depths *m*_*ωm*_ and phase shifts *ϑ*_*ωm*_ and the calculated *m*_*ωc*_ and *ϑ*_*ωc*_ was minimized by finding optimal values for lifetimes *τ*_*i*_ and fractions *α*_*i*_[13].

(1) $$\vartheta_{\mathrm{\omega c}}=\arctan\frac{\sum_{i}\frac{a_{i}\omega \tau_{i}}{1+{\left(\omega \tau_{i}\right)}^{2}}}{\sum_{i}\frac{a_{i}}{1+{\left(\omega \tau_{i}\right)}^{2}}}$$

(2) $$m_{\mathrm{\omega c}}=\sqrt{{\left(\sum_{i}\frac{a_{i}}{1+{\left(\omega \tau_{i}\right)}^{2}}\right)}^{2}+{\left(\sum_{i}\frac{a_{i}\omega \tau_{i}}{1+{\left(\omega \tau_{i}\right)}^{2}}\right)}^{2}}$$

Error function was given by:

(3) $$x^{2}={\sum_{\omega }\left(\vartheta_{\mathrm{\omega m}}-\vartheta_{\mathrm{\omega c}}\right)}^{2}+{\sum_{\omega }\left(m_{\mathrm{\omega m}}-m_{\mathrm{\omega c}}\right)}^{2}$$

Measurements were made in two independent experiments using 50 nM F_2_FlAsH in the presence or absence of either 60 nM βγM or αolf and α13 (with or without 10 uM GTPγS) or 600 nM of tetracysteine-labeled peptide (FLNCCPGCCMEP). F_2_FlAsH and its complexes were preincubated at 28°C for 6 h before lifetime measurements at room temperature.

### Spectrophotometric measurements of F_2_FlAsH-complexes

Fluorescence emission spectra of free F_2_FlAsH (5 nM) and its complexes with G-proteins (12.5 nM αolf, with or without 10 μM GTPγS, or 15 nM βγM subunits) were determined using a Perkin-Elmer LS 55 luminescence spectrometer (PerkinElmer Inc, Waltham, MA, USA) with excitation at 480 nm and a 10 nm emission slit width at 100 nm/min scan speed.

### Fluorescence anisotropy measurements

All fluorescence anisotropy measurements were carried out at 28°C in 96-well half area microtiter plates (Corning Product No.3993, Corning Life Sciences, Lowell, MA, USA) in a Pherastar platereader (BMG LABTECH GmbH, Ortenberg, Germany) [14]. Fluorescence anisotropies were measured using (polarized) excitation at 485 nm (20 nm bandwidth) and simultaneous dual (polarized) emission at 520 nm (20 nm bandwidth), which enabled recording of fluorescence emission intensities that are parallel and perpendicular to the plane of excitation light. Erythrosin B was used for fluorescence polarization calibration [15].

Measurements were conducted in duplicate or quadruplicate in two or three independent experiments. The apparent affinities of GDP, GMP, GTPγS and GppNHp were determined by their abilities to modulate F_2_FlAsH fluorescence anisotropy in the presence of αβγM heterotrimers. For experiments with nucleotide-depleted αi-heterotrimers, 5 nM F_2_FlAsH was used to label approximately 40 nM G-proteins and nucleotide affinities were determined at 2 h from the start of the experiment. For experiments with αq, αs_long_, αs_short_, αolf and α13, 12.5 nM α-subunits were preincubated on ice with 15 nM βγM for 60 min, to allow the subunits to associate, before addition of 15 nM F_2_FlAsH and nucleotides. Nucleotide affinities for αs_long_ + βγM, αs_short_ + βγM, αq + βγM and αolf + βγM were determined at 6 h from the start of the experiment and for α13 + βγM at 14 h. All measurements (unless specified otherwise) were made in 20 mM HEPES buffer, pH = 7.8, with 1 mM EDTA, 2 mM MgCl_2_, 10 mM NaCl, 2 mM β-mercaptoethanol, 1 mM TCEP and 0.1% C12E10. All reagents, proteins and microtiter plates were kept on ice prior to the initiation of the measurements. In experiments where F_2_FlAsH and G-proteins were subjected to heat denaturation, microtiter plates that had been measured in the platereader for 6 h were sealed with AbsorbMax adhesive film (Excel Scientific Inc, Victorville, CA, USA) and heated at 70°C for 1 h in an Eppendorf Thermomixer Comfort (Eppendorf AG, Hamburg, Germany) with shaking at 400 rpm. The plates were then cooled to room temperature, unsealed and remeasured in the platereader. Data was then collected at 30 min from the start of the measurement.

### Data analysis

**Figure 1:**
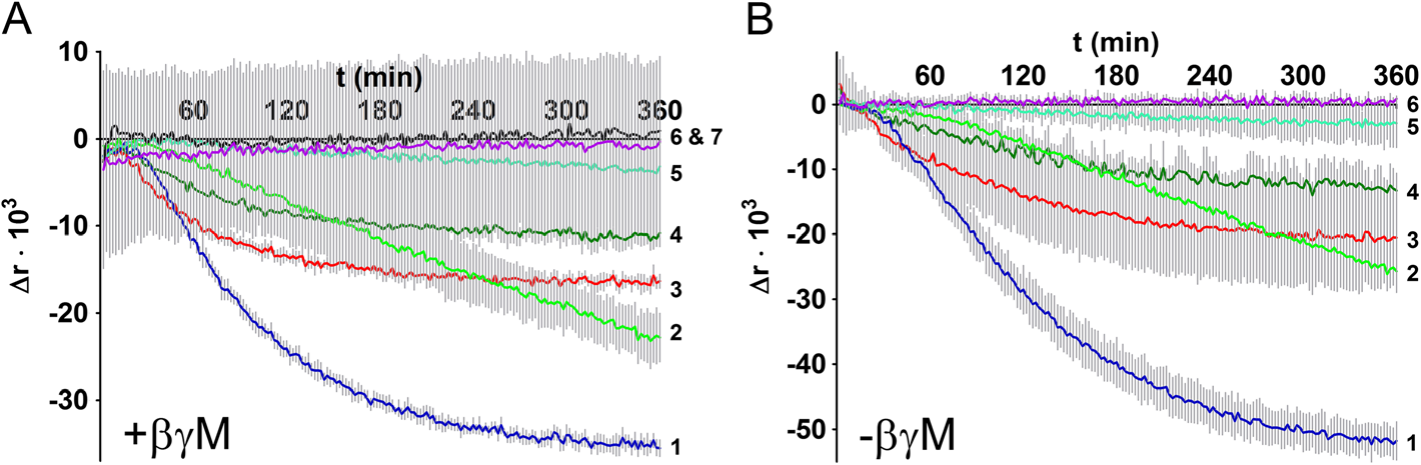
**The effect of 10 μM GTPγS on the formation of complexes between F**_**2**_**FlAsH and G-proteins in the presence (A) or absence of βγM subunits (B).** Data are presented as the time-dependent attenuation of fluorescence anisotropy in comparison to GTPγS-untreated F_2_FlAsH-G-protein complexes. The preparations used were αolf (1), αq (2), αs_short_ (3), αs_long_ (4), α13 (5), none (6) and βγM (7). Data is from two or three independent experiments carried out in duplicate and presented as mean ± SD as error bars.

All data were analyzed using nonlinear regression with GraphPad Prism 5.0 (GraphPad Software Inc., La Jolla, CA, USA. Fluorescence anisotropy data was baseline (F_2_FlAsH-G-protein complex with no added nucleotides) corrected for each experiment before data was pooled and fitted for determination of nucleotide affinities. Kinetic curves (Figure 1) were also baseline corrected to show the appearance of nucleotide-sensitivity. Apparent affinity values (logEC_50_) were calculated using three parameter competitive binding equations (Y = Bottom + [(Top-Bottom)/(1 + 10^^(X-LogEC^_50_^)^)]), where X corresponds to logarithm of molar concentration of nucleotide.

## Results and discussion

### Fluorescence intensities and lifetimes of F_2_FlAsH-complexes

The fluorescence lifetime and quantum yield of fluorophores can be altered by interactions with their molecular environment. As the fluorescence intensity of F_2_FlAsH was increased up to twofold by the addition of G-protein α-subunits – with no significant shift in emission or absorbance maxima in comparison to free F_2_FlAsH (Additional file 1: Figure S1) - we decided to investigate whether this increase in fluorescence intensity would also be reflected in alterations of the fluorescence decay rates of F_2_FlAsH complexes, as higher fluorescence intensities suggest higher quantum yields, with proportionally longer lifetimes of fluorescence decay.

We measured the effects of a tetracysteine-labeled peptide, βγM, α13 and αolf (with or without 10 μM GTPγS) on the fluorescence lifetimes of F_2_FlAsH (Table 1, Additional file 2: Figure S2). Free F_2_FlAsH exhibited a two component exponential decay rate with a shorter component of 1.0 **±** 0.2 ns and a longer component of 4.3 **±** 0.1 ns, with the shorter lifetime component comprising 34 **±** 3% of the signal. Addition of GTPγS to free F_2_FlAsH did not significantly alter fluorescence decay rates or their relative proportions. Addition of a tetracysteine labeled peptide however decreased the proportion of the faster decaying component down to 11 **±** 2%, as did the addition of βγM, which decreased the 1.0 ns component down to 25 **±** 2% of the total. When αolf subunits were added to F_2_FlAsH the proportion of the faster decaying component was decreased to 12 **±** 5% in the absence of GTPγS, but when the nucleotide was present we observed a fluorescence decay rate that was best described by a 3-component fit with a previously undetected very rapidly decaying component (τ < 0.1 ns, 17% of the signal), while the proportion of the 1.0 ns component was increased to 31%. Addition of α13, which had very low sensitivity to nucleotide-dependent changes in fluoresence anisotropy, also had limited effects of the fluorescence decay rates of F_2_FlAsH: the 1.0 ns component was not greatly altered at 37 **±** 4% (in comparison to free F_2_FlAsH, where this component was at 34 **±** 3%) in the absence of GTPγS and was at 38 **±** 3% in the presence of the nucleotide. No rapid 0.1 ns component was detected in the presence of GTPγS for α13.

**Table 1:** **Fractions of F**_**2**_**FlAsH and its complexes’ fluorescence with a lifetime of 1 ns**

	**Fraction τ**_**(1.0 ns)**_^**a **^**%**	**χ**^**2**^
F2FlAsH	34 ± 3	1.9
F2FlAsH + GTPγS	29 ± 1	3.7
CCPGCC-peptide	11 ± 2	3.8
βγM	25 ± 2	4.6
αolf	12 ± 5	1.67
αolf + GTPγS	56 ± 4^b^	12^b^
α13	37 ± 4	6.7
α13 + GTPγS	38 ± 3	2.5

^a^Fluorescence lifetimes were fitted to equations (1–3) with two components (1.0 ± 0.2 ns and 4.3 ± 0.1 ns) and fractions corresponding to 1.0 ns are reported as mean ± SD.

^b^A better fit was obtained by including a third very fast component corresponding to 17 % of fluorescence, but with a lifetime that was below the measurement range of the instrument (τ < 0.1 ns).

These results indicate that the nucleotide-dependent changes in fluorescence anisotropy, which will be described in the following paragraphs, could be the result of quenching of F_2_FlAsH by guanine nucleotides that bind to G-protein α-subunits in close proximity to the fluorophore. This quenching would (by decreasing the time available for rotation) increase fluorescence anisotropy. However, if the reaction is accompanied by a change in the rotational correlation time (as a result of altered binding or conformational changes) it would have the opposite effect on steady state fluorescence anisotropy measurements. Thus, the final results would depend on the extent of changes in fluorescence lifetime in comparison to changes in the rotational correlation time.

### The effects of G-proteins and nucleotides on the fluorescence anisotropy of F_2_FlAsH

**Figure 2:**
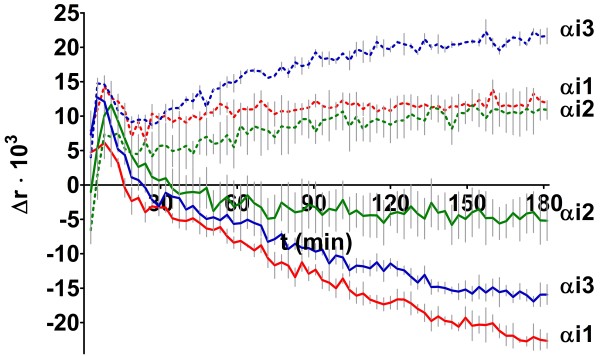
**The effect of 10 μM GTPγS (solid lines) and 10 μM GDP (dotted lines) on the formation of complexes between F**_**2**_**FlAsH and Gi-protein heterotrimers.** Data are presented as the time-dependent change in fluorescence anisotropy in comparison to nucleotide-untreated F_2_FlAsH-Gi-protein complexes. Data is from two or three independent experiments carried out in duplicate and presented as mean ± SD as error bars.

We also investigated the kinetics and nucleotide-dependence of F_2_FlAsH binding to various G-protein subunits by using fluorescence anisotropy. When purified tetracysteine-tagged βγM-subunits were added to F_2_FlAsH, a time-dependent increase in fluorescence anisotropy of F_2_FlAsH was seen. The fluorescence anisotropy of free F_2_FlAsH or F_2_FlAsH-βγM complexes was not significantly affected by the presence or absence of GTPγS (Figure 1 lines 6 and 7). Addition of purified α-subunits to F_2_FlAsH (in the presence of βγM-subunits) caused an additional increase in fluorescence anisotropy in comparison to F_2_FlAsH-labeled βγM. The magnitude of the increase depended on the α-subunit subtype and this increase in fluorescence anisotropy could be attenuated by the addition of 10 μM GTPγS for all 8 α-subunits studied (Figure 1, Figure 2 solid lines).

When F_2_FlAsH was added to purified wild type α-subunits in the absence of tetracysteine-tagged βγM subunits, the large GTPγS-sensitive signal was still evident (Figure 1B) and this time-dependent increase in nucleotide-sensitive fluorescence anisotropy was practically unaltered by the addition of βγM-subunits to the mixture (Figure 1A), except for αolf, which had the largest nucleotide-sensitive signal amplitude and this was reduced in the presence of βγM (Figure 1 line 1). These results indicate that F_2_FlAsH interacts directly with G-protein α-subunits and nucleotide-dependent changes in anisotropy are not the results of α-subunit binding to F_2_FlAsH-labeled βγM subunits in a way that alters the latter’s fluorescent properties.

**Figure 3:**
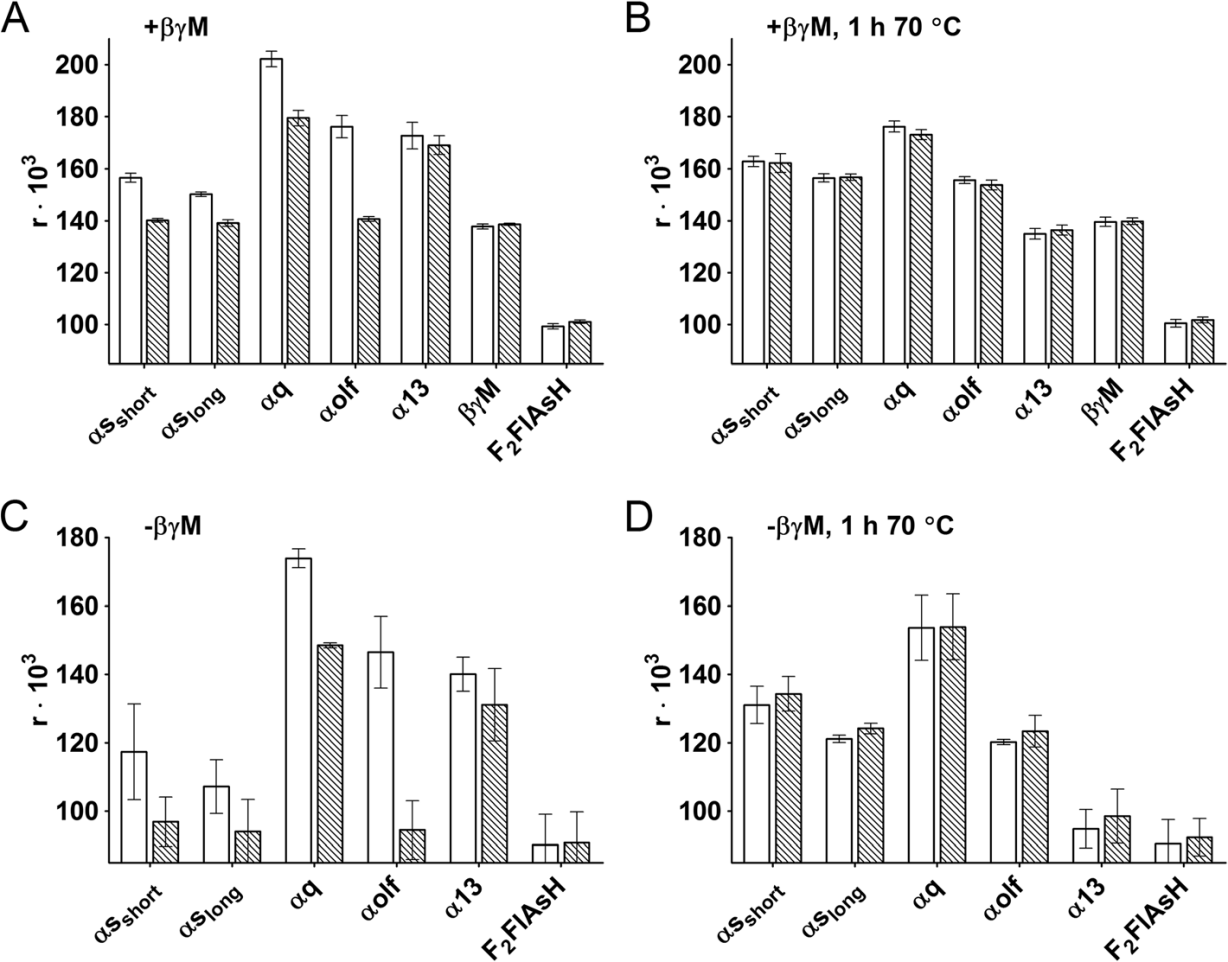
**Effect of GTPγS on the fluorescence anisotropy of F**_**2**_**FlAsH-G-protein preparations.** The fluorescence anisotropy of α-subunits with **(A, B)** or without **(C, D)** βγM subunits, in the absence (solid bars) or presence of 10 μM GTPγS (striped bars), was measured after 6 h of incubation at 28°C **(A and C)** and again after subsequent heat treatment for 1 h at 70°C, **(B and D)**. Data is from two independent experiments carried out in duplicate, presented as mean ± SD as error bars.

In the case of βγM complexes with αs_short_, αs_long_ and αolf, addition of GTPγS depressed the fluorescence anisotropy signal close to F_2_FlAsH-labeled βγM-levels (Figure 3A), which we interpret (based of fluorescence lifetime measurements) as possible quenching of the fluorophore by the guanine nucleotide in close-proximity to the binding site of F_2_FlAsH, or possibly also by some conformational rearrangements of the G-protein heterotrimers (or just α-subunits) that made the F_2_FlAsH-binding sites on α-subunits less favorable for F_2_FlAsH interactions upon nucleotide binding. These conformational rearrangements may have even led to complete loss of binding for some α-subunits, although the appearance of a sub 0.1 ns fluorescence lifetime component (Table 1) in the presence of GTPγS would not be explained by this. Additionally: as G-protein α-subunits may aggregate under certain conditions [16], it is possible that nucleotide-dependent changes may have been caused by dissociation of α-subunit oligomers (GTPγS is thought to disaggregate α-subunits). These oligomers could have presented multiple cycsteine residues to F_2_FlAsH in close proximity (in the absence of added nucleotides), so F_2_FlAsH could have cross-linked these α-subunits (leading to large increases in fluorescence anisotropy).

Large nucleotide-sensitive changes in fluorescence anisotropy for the aforementioned α-subunits were also observed in the absence of βγM-subunits, where addition of nucleotides depressed the signal close to free F_2_FlAsH levels (Figure 3C), indicating that if there are any energy transfer processes between the F_2_FlAsH-labeled α- and βγM-subunits, they do not contribute significantly to the observed signals.

In contrast to αs_short_, αs_long_ and αolf the fluorescence anisotropy remained higher for αq and α13 in the presence of GTPγS (Figure 3A, 3C), when compared to F_2_FlAsH-βγM or F_2_FlAsH. This was the case in experiments done both in the presence or absence of βγM. This seems to indicate that F_2_FlAsH was still able to associate with αq and α13 in their nucleotide-bound states - but as this interaction with F_2_FlAsH had limited nucleotide sensitivity the F_2_FlAsH-binding site on these subunits is probably not close enough or oriented properly for quenching (or other mechanisms that lower fluorescence anisotropy such as disaggregation or conformational rearrangements) by guanine nucleotides. Additionally: low nucleotide sensitivity could also be caused by slower nucleotide binding to αq and α13 (Figure 1 lines 2 and 5).

**Figure 4:**
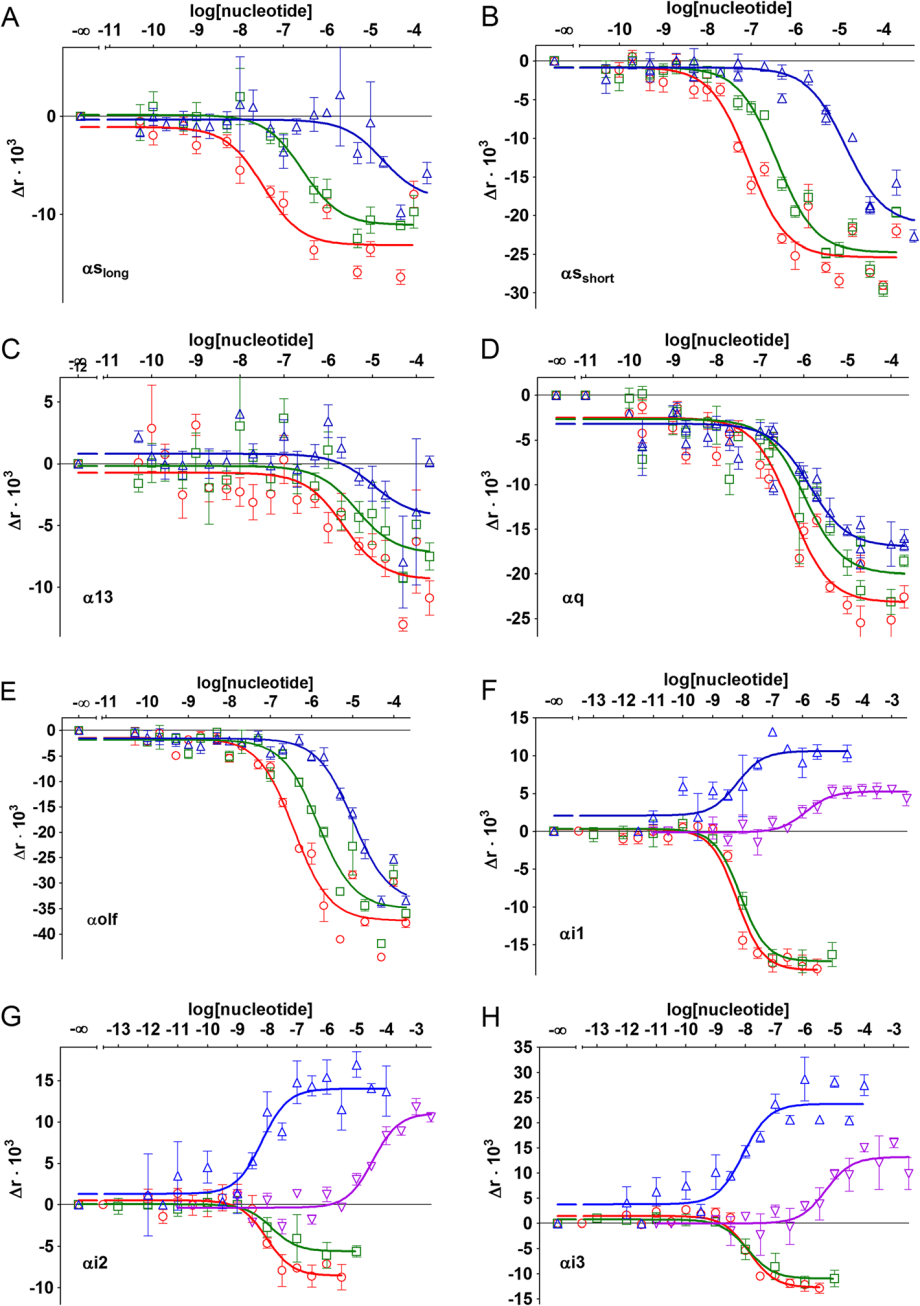
**Concentration-dependent effects of nucleotides on the fluorescence anisotropy of F**_**2**_**FlAsH-αβγM complexes.** The data was collected after incubation of the complexes with different concentrations of GTPγS (red circles), GppNHp (green squares), GDP (blue pyramids) or GMP (magenta triangles). Measurements were taken at different timepoints for the various α-subunits: **A** - αs_long_ at 6 h; **B** - αs_short_ at 6 h; **C** - α13 at 14 h; **D** - αq at 6 h; **E** - αolf at 6 h; **F** - αi1 at 2 h; **G** - αi2 at 2 h; and **H** - αi3 at 2 h. Data is from two or three independent experiments in duplicate, presented as mean ± SD as error bars.

GTPγS also caused a time-dependent decrease of fluorescence anisotropy with F_2_FlAsH-αi-subunit complexes (Figure 2 solid lines). Conversely to the other 5 α-subunits studied, when GDP was added to these nucleotide depleted F_2_FlAsH-αiβγM complexes, an increase in fluorescence anisotropy was seen (Figure 2, dotted lines). For the other 5 α-subunit subtypes studied GDP decreased the fluorescence anisotropy of their F_2_FlAsH complexes, similarly to GTPγS, although to a lesser extent (Figure 4A-E).

There were also some differences between the three αi-subtypes themselves: F_2_FlAsH complexes with αi2 had smaller nucleotide-sensitive signal amplitude than with αi1 or αi3. This could indicate that there are specific differences between the interaction of F_2_FlAsH and the αi2 subunit, when compared to αi1 and αi3. Alternatively this effect could be caused by differences in protein composition, which was evaluated by using Ag-stained SDS-PAGE gels (as described in [11]). This could have resulted in under- or overestimation of G-protein α-subunit concentrations in the purified protein preparations of different αi-subtypes, even though the samples were resolved and stained in parallel and had very similar compositions. Due to this uncertainty, no direct comparison between F_2_FlAsH-βγM and GTPγS-treated and untreated F_2_FlAsH-αiβγM complexes was undertaken, as had been for the other 5 α-subunit preparations (Figure 1A, Figure 3A).

The fluorescence anisotropy of αs_short_, αs_long_, αq and αolf, αi1, αi2 and αi3 complexes with F_2_FlAsH all yielded robust changes caused by the addition of guanine nucleotides (Figure 1, Figure 2). In comparison the nucleotide-dependent fluorescence anisotropy signal of F_2_FlAsH-α13 complexes (in the presence or absence of βγM) was very small and it appeared very slowly (Figure 1 line 5). So even after 14 h of incubation at 28°C only a small effect of nucleotides on the fluorescence anisotropy could be detected, but longer incubation times could not be applied due to evaporation of the solvent. Similarly slow emergence of nucleotide-dependent effects was observed for F_2_FlAsH-αq complexes, but this signal had higher amplitude (Figure 1, line 2). This is consistent with previously published results as both α13- and αq-subunits have been found to have slow GDP release rates and to require high concentrations of GTPγS for activation [17]. The nucleotide-sensitive signal of the αolf- F_2_FlAsH complex, which had the highest amplitude, stabilized within 6 h - while the nucleotide-sensitive changes in F_2_FlAsH-αs_short_ or F_2_FlAsH-αs_long_ complexes reached their maximum values in approximately 3 h.

Nucleotide-sensitive changes in F_2_FlAsH fluorescence anisotropy appeared rapidly for αi-subunit preparations as well (Figure 2), although they were assayed at higher concentrations (~40 nM αiβγM heterotrimer) and in an approximately 8-fold stoichiometric excess when compared to F_2_FlAsH (5 nM in the αi-subunit assays). This seemed to indicate that nucleotide-dependent F_2_FlAsH binding to G-protein α-subunits could not be easily displaced by an apparent excess of βγM-subunits, even though the tetracysteine-tag on βγM has a very high affinity for FlAsH-analogues and would be expected to bind nearly all of the F_2_FlAsH present. This should make the fluorophore unavailable for presumable less favorable interactions with α-subunits, as the latter do not contain engineered tetracysteine motifs.

### The sensitivity of F_2_FlAsH-G-protein complexes to their molecular environment: indications of As-cysteine interactions

F_2_FlAsH could interact with G-protein α-subunits through either nonspecific hydrophobic interactions or through more specific binding modes such as arsenic-thiol interactions. Our results support the latter and indicate that G-protein α-subunits compete with high affinity for F_2_FlAsH binding with other cysteine-rich proteins or dithiol motifs present in the reaction medium. For example, the presence of 2 mM β-mercaptoethanol in the assay buffer (in comparison to experiments done in the presence of 5 μM β-mercaptoethanol) had only a small effect on nucleotide-sensitive changes in fluorescence anisotropy. This indicates that F_2_FlAsH interactions with G-protein α-subunits are not easily displaced by monothiols. We also tested whether the interaction of F_2_FlAsH with G-protein α-subunits could be blocked with dithiols. In this case no increase (compared to free F_2_FlAsH) in fluorescence anisotropy or any GTPγS-sensitivity changes could be detected when F_2_FlAsH was added to G-proteins in the presence of 200 μM ethanedithiol, which indicates that the F_2_FlAsH-α-subunit interactions were completely blocked by this reagent. This result seems to indicate that F_2_FlAsH binds multiple cysteine residues on G-protein α-subunits. As no such residues are present in the highly conserved guanine nucleotide binding site of G-proteins [18], it appears that F_2_FlAsH acts as an allostering sensor of nucleotide binding. We also tested whether the F_2_FlAsH-α-subunit interaction could be blocked by the addition of arsenous acid (100 μM hydrolyzed AsCl_3_) and found that in this case the nucleotide-dependent fluorescence anisotropy signal was attenuated, but not completely lost. This indicates that biarsenical ligands have a higher affinity for cysteine residues on G-protein α-subunits than arsenic itself. Unfortunately we were not able to directly determine the affinity of F_2_FlAsH for G-protein α-subunits as a nonfluorescent biarsenical ligand was not available to us for measuring nonspecific F_2_FlAsH binding.

We also tested whether nonfluorinated FlAsH (Lumio Green) would associate in a nucleotide-sensitive manner with G-protein α-subunits (αs_short_, αs_long_, αq, αolf and α13). The results were comparable to experiments done using F_2_FlAsH, indicating that the formation of nucleotide-sensitive complexes with G-protein α-subunits is not a unique property of F_2_FlAsH but a more general interaction with biarsenical ligands. We also tested whether fluorescein could associate with G-proteins in a nucleotide-dependent manner (presumably through hydrophobic interactions): no changes in fluorescence anisotropy caused by the addition of G-proteins could be detected; neither could we detect any nucleotide effects (data not shown). This further supports the hypothesis that FlAsH analogues bind to cysteine residues on α-subunits via high affinity arsenic-thiol interactions.

Of course it is probable that nucleotide-sensitive FlAsH and F_2_FlAsH binding is not an effect that is unique to these eight G-protein α-subunits tested. Instead similar effects might be found for other proteins that have multiple cysteine residues near structural motifs that undergo conformational rearrangements that alter their interaction with biarsenical ligands or bring the fluorophores in close proximity with quenching moieties. If this is so then FlAsH-analogues could be applied as an allosteric probe do study the functioning of such proteins *in vitro*.

Heterotrimeric G-proteins are known to be thermolabile [19,20] so we could test whether disruption of their active conformational state would have an effect on their nucleotide-dependent interaction with F_2_FlAsH. When F_2_FlAsH-G-protein complexes were heated at 70°C for 1 h after 6 h of measurements at 28°C, differences between the fluorescence anisotropy signals of nucleotide-treated and untreated F_2_FlAsH-G-protein complexes were lost (Figure 3B, 3D). However, the fluorescence anisotropy of most F_2_FlAsH-G-protein complexes, with the exception of α13, was not decreased down to the levels of free F_2_FlAsH (Figure 3D) or F_2_FlAsH-βγM (Figure 3B) by the heat-treatment, indicating incomplete dissociation of the F_2_FlAsH-α complexes. This suggests that some interactions of the thermally denatured α-subunits with F_2_FlAsH remained for most of them even after nucleotide binding capability was lost, which could be explained by F_2_FlAsH binding to α-subunit cysteine residues, or possibly by some nonspecific interactions of F_2_FlAsH with the denatured proteins. For some α-subunits (αs_short_, αs_long_) the absolute fluorescence anisotropy values even increased after heat treatment, which could indicate increased exposure (or more favorable binding geometry) of cysteine residues that were able to associate with F_2_FlAsH on these α-subunits after denaturation. Nucleotide-dependent changes in fluorescence anisotropy could also be abolished for αi-heterotrimers by heat-treatment, but no direct comparison to F_2_FlAsH-βγM was undertaken as the composition of these samples was not determined quantitatively and thus we could not be certain that identical amounts of the relevant G-protein subunits were compared.

The influence of other, non-specific proteins on the nucleotide sensitivity of F_2_FlAsH-αβγM complexes (tested with αi3βγM) was measured in the presence of BSA or pyruvate kinase. BSA caused a 50% increase in F_2_FlAsH-fluorescence intensity and (in both the absence and presence of αi3βγM), but no such increase was detected when pyruvate kinase was used as a non-specific protein even at maximal concentrations (100 μg/ml) tested. Both absolute levels of fluorescence anisotropy and nucleotide-dependent effects were unaltered by pyruvate kinase. BSA increased absolute anisotropy by about 30% and this was accompanied by complete disappearance of nucleotide-dependent effects with an EC_50_ of about 0.5 μg/ml, which for this assay was in the same range as the concentration of the G-protein α-subunit itself.

There are multiple reports in the literature that BSA binds fluorescein analogues with high affinity [21], while for pyruvate kinase no such reports were found. This attenuation of nucleotide-sensitivity by BSA indicates that the F_2_FlAsH fluorophore is either relatively exposed to the solvent in the F_2_FlAsH-αi3βγM complex and nonspecific interactions with BSA (which contains multiple cysteine residues) can completely block the sensitivity of F_2_FlAsH fluorescence anisotropy to G-protein α-subunits in their various nucleotide bound states. Alternatively the interaction between F_2_FlAsH and BSA is of a comparable or higher affinity than the interaction between F_2_FlAsH and G-protein α-subunits, thus limiting the availability of F_2_FlAsH for forming a fluorescent complex with the α-subunits.

### Characterization of the nucleotide sensitivity of F_2_FlAsH-G-protein complexes

As guanine nucleotides had robust effects on the fluorescence anisotropy of F_2_FlAsH-αβγM complexes we utilized this system for the characterization of nucleotide binding to G-proteins. We note that due to slow nucleotide-exchange kinetics and low nucleotide-affinities of some α-subunits, traditional orthosteric ligands (labeled nucleotides) would have been limited in their applicability, whereas F_2_FlAsH as an allosteric probe could be used to monitor their nucleotide-bound states without being as limited by a nucleotide exchange requirement.

Eight different α-subunits in combination with F_2_FlAsH and βγM were studied to reveal the effects of GDP, GMP, GTPγS and GppNHp on these complexes. Depending on the α-subunit, large differences in signal amplitudes, nucleotide affinities and even in the type of the effect (increase or decrease of fluorescence anisotropy) upon addition of guanine nucleotides, was seen (Figure 4).

The four nucleotides tested had a similar order in their apparent binding affinities for all of the G-proteins studied, with GTPγS having the highest and GMP the lowest affinity (Table 2). GDP was equipotent with GTPγS and GppNHp for αi-complexes, but less potent for all other G-protein preparations tested. Similar trends were seen for the amplitude of the nucleotide-dependent change in fluorescence anisotropy, with GTPγS causing the biggest change, while for GDP and GMP the changes were smaller for most α-subunits (with the exception of αi2 and αi3). This could indicate that GTPγS binding to α-subunits can cause a conformational rearrangement that brings the quenching guanine moiety closer to the F_2_FlAsH binding site than either GDP or GMP - or alternatively: GTPγS leads to disaggregation of α-subunit oligomers while GDP and GMP cause smaller conformational rearrangements.

**Table 2:** **Apparent affinities of nucleotides in F**_**2**_**FlAsH-αβγM complexes**

	**pEC**_**50**_^**a**^			
	**GTPγS**	**GppNHp**	**GDP**	**GMP**
αs_long_	7.47 ± 0.09	6.56 ± 0.07	4.72 ± 0.18	ND
αs_short_	7.06 ± 0.04	6.42 ± 0.04	4.85 ± 0.05	< 3
α13	5.65 ± 0.13	5.36 ± 0.14	4.98 ± 0.23	ND
αq	6.27 ± 0.05	5.96 ± 0.07	5.87 ± 0.06	< 3
αolf	6.43 ± 0.04	5.84 ± 0.05	5.00 ± 0.03	< 3
αi1	8.19 ± 0.05	8.08 ± 0.04	8.19 ± 0.16	5.89 ± 0.12
αi2	8.02 ± 0.09	7.84 ± 0.14	8.17 ± 0.13	4.45 ± 0.07
αi3	7.87 ± 0.06	7.93 ± 0.07	8.08 ± 0.10	5.29 ± 0.13

^a^negative logarithm of concentration of the nucleotide causing 50% of fluorescence anisotropy changes of F_2_FlAsH-αβγM complexes (mean ± SD), determined after 6 h incubation for αs_long_, αs_short_, αq and αolf, after 14 h for α13, after 2 h for αi1, αi2 and αi3.

Data is from two to three independent experiments carried out in duplicate. Data were pooled and then fitted to three parameter competitive binding equations.

Gi-proteins differed from the other five G-protein subtypes tested in several ways and there were also some specific differences between the αi-subunits themselves: these three subunits (purified in nucleotide-depleting conditions) exhibited striking differences between the direction of the effects of activating guanine nucleotides (nonhydrolyzable GTP-analogues) and GDP or GMP (thought to preferentially bind to and stabilize inactive G-protein conformations). In comparison to experiments done without added nucleotides, the addition of GDP and GMP caused an increase in fluorescence anisotropy, whereas the addition of GTPγS and GppNHp resulted in a decrease (Figure 4F-4H). This could indicate that the nucleotide-depleted αi-subunit preparations partially denatured at 28°C and could no longer interact with F_2_FlAsH (when no additional GDP or GMP was present), whereas the addition of GDP or GMP could stabilize the nucleotide-free pool of α-subunits and preserve their F_2_FlAsH binding ability, thus increasing the signal. Rapid degradation of nucleotide-depleted αi-subunits at 28°C is also consistent with our previous measurements, where these nucleotide-depleted protein preparations lost their ability to bind Bodipy-FL-GTPγS quickly (τ_½_ = 20 ± 5 min) at 28°C [11], but addition of saturating amounts of nucleotides could stabilize the complex. Alternatively the stabilizing effects of GDP and GMP could be connected with the stability of the G-protein heterotrimer: GDP-bound αi-subunits would be expected to be associated with βγM-subunits, which would reduce their rotational mobility, while GTPγS treatment would be expected to dissociate the heterotrimer and lead to a decrease in fluorescence anisotropy. Disaggregation of αi-subunits by GTPγS is also a possible mechanism behind the different effects of activating and nonactivating guanine nucleotides as αi subunits are thought to be especially likely to aggregate [16]. Additionally, as we had determined affinity of these three Gi protein preparations for GDP and GTPγS previously using a Bodipy-FL-GTPγS-based nucleotide displacement assay [11] in a similar environment (except no TCEP was added to the buffer in the Bodipy-FL-GTPγS assay), we could compare the results from the two methods: the affinity of αiβγM complexes for GDP and GTPγS determined using Bodipy-FL-GTPγS was in close agreement with the values obtained by using the F_2_FlAsH-based assay. The nucleotide-depleted αiβγM preparations also had the highest nucleotide affinities of all the G-protein preparations tested (Table 2).

αi-subunits also exhibited specific differences in the relative amplitudes of nucleotide effects: F_2_FlAsH complexes of αi2 and αi3 exhibited a greater change in fluorescence anisotropy upon addition of GDP, instead of GTPγS (Figure 4G, 4H). This could reflect the large stabilizing effect of GDP on the nucleotide-free α-subunit pool in those protein preparations as the relatively smaller effect GDP and GMP had on the αi1-subunit could be caused by its resistance to nucleotide depletion during protein purification, so the αi1-protein preparation had a greater GDP content. This would explain why there seemed to be less degradation of the nucleotide depleted F_2_FlAsH-αi1-protein complex in the absence of added nucleotides.

In contrast to αi-subunits, all of the other α-subunits tested exhibited similar effects in complexes with F_2_FlAsH for GTPγS, GppNHp, GDP and GMP: a decrease in fluorescence anisotropy (Figure 4F-4H), indicating increased quenching of the F_2_FlAsH-fluorphore or possibly a change in α-subunit conformation/aggregation that makes them less accessible to F_2_FlAsH upon nucleotide binding. For αi-subunits the reverse was true and GDP and GMP seemed to stabilize the F_2_FlAsH-G-protein interaction.

There may be several reasons why the effects GDP and GMP had on αi-subunits differed from other G-protein subtypes. One reason may be that αs_short_, αs_long_, αq, αolf and α13-proteins were not purified in nucleotide-depleting conditions, so they could have been saturated with GDP. These 5 α-subunits had all been purified using GST-Ric8 association [17] instead of StrepII-labeled γ2-subunits, as was the case for αi-heterotrimers. So these two purification approaches may have yielded protein preparations that had significantly different compositions in terms of nucleotide and cofactor content and also in G-protein subunit stoichiometry: tandem affinity chromatography would be expected to yield αi-subunits at up to equimolar concentrations with βγM as each immobilized βγM-subunit can bind up to one α-subunit. Whereas when the GST-Ric8-purified α-subunits (12.5 nM, determined by the manufacturer) were reconstituted with purified βγM subunits, the possibility existed that they could have been present in a stoichiometric excess when compared to βγM, which we estimate should have been present at approximately 15 nM (based on UV-absorbance and analysis of Ag-stained SDS-PAGE gels). Control experiments with 15 nM F_2_FlAsH, 12.5 nM αq and 30 nM βγM did not, however, yield significantly different nucleotide effects on F_2_FlAsH-αqβγM fluorescence anisotropy.

αiβγM preparations were also assayed at higher protein and lower F_2_FlAsH concentrations than αs_long_, αs_short_, αq, αolf and α13. The influence of the stoichiometry of F_2_FlAsH-labeling (ratio of F_2_FlAsH to G-protein heterotrimers) on nucleotide-dependent changes in fluorescence anisotropy was investigated using αqβγM preparations (2 to 20 nM F_2_FlAsH and 12.5 nM αq and 15 nM βγM) and found to mainly affect the signal to noise ratio of the assay, while having little effect on the amplitude of the nucleotide-dependent change in fluorescence anisotropy. This suggests that the nucleotide binding experiments done with αs_short_, αs_long_, αq, αolf and α13 could be similarly representative of their nucleotide binding affinities (in comparison to experiments with labeled nucleotides) as those done using αiβγM preparations, even though the subunit stoichiometries and F_2_FlAsH to G-protein ratios were not identical for all of the F_2_FlAsH-G-protein complexes studied. Indeed, our estimation of the apparent affinity of GTPγS for αs-subunits is close to previously published values determined using assays based on the displacement of fluorescent nucleotides [22] and high concentrations (10–30 μM) of GTPγS have previously been found necessary to activate αq and α13 [17], which is reflected by our results: these two proteins had the lowest apparent affinity for GTPγS out of all the G-protein subtypes studied.

When comparing the effects of nucleotides on F_2_FlAsH-G-protein complexes, it was apparent that F_2_FlAsH-αiβγM protein complexes had the highest fluorescence anisotropy in the presence of GDP or GMP, while the same was true for F_2_FlAsH-αs_short_, αs_long_, αq, αolf and α13 complexes in the absence of any added nucleotides. It has been suggested that αi-subunits in their GDP-bound state could have multiple binding sites for βγ-subunits [23] (and are likely to aggregate themselves [16]), which could also (in addition to inhibition of denaturation of nucleotide-free αi-subunits) explain the increase in fluorescence anisotropy upon GDP and GMP binding to αi-subunits: βγM subunits were probably present at a slight excess in the αiβγM protein preparations, so they could form complexes with at least some αiβγM-heterotrimers. The added mass of a second βγM binding to the G-protein heterotrimer would be expected to further decrease the rotational mobility of the F_2_FlAsH-G-protein complex and result in a slightly higher anisotropy. Thus absence of any GDP or GMP-induced increase in the fluorescence anisotropy of αs_short_, αs_long_, αq, αolf and α13 complexes with F_2_FlAsH and βγM could be explained by the lack of a second βγM-subunit binding site on these α-subunits. Alternatively the effects of GDP or GMP on fluorescence anisotropy of αiβγM complexes could be explained by interactions with some regulatory proteins that were copurified alongside the heterotrimers using βγM-tandem affinity chromatography, but not with GST-Ric8-purified α-subunits.

## Conclusions

F_2_FlAsH bound to G-protein α-subunits allosterically via As-thiol interactions. The presence or absence of guanine nucleotides had an effect on the fluorescence anisotropy, intensity and lifetime of F_2_FlAsH-G-protein complexes. F_2_FlAsH was sensitive to nucleotide binding to αi1, αi2, αi3, αs_short_, αs_long_, αq and αolf, but showed low sensitivity to nucleotide binding to α13. Nucleotide affinities (as determined by changes in the fluorescence anisotropy of F_2_FlAsH-G-protein complexes) for GTPγS, GppNHp, GDP and GMP were comparable to values obtained in assays based on the displacement of fluorescently labeled nucleotides [11]. GDP and GMP seemed to stabilize nucleotide-depleted F_2_FlAsH-αi-subunit complexes, whereas for all other α-subunits tested the fluorescence anisotropy decreased in the presence of these nucleotides. GTPγS and GppNHp decreased fluorescence anisotropy for F_2_FlAsH-α-subunit complexes for all G-protein subtypes studied.

## Abbreviations

G-protein: Guanine nucleotide binding protein; GPCR: G-protein coupled receptor; GDP: Guanosine diphosphate; GMP: Guanosine monophosphate; GTPγS: Guanosine 5′–O-[gamma-thio]triphosphate; GppNHp: Guanosine 5′-[β,γ-imido]triphosphate; C12E10: Polyoxyethylene (10) lauryl ether; TCEP: tris(2-carboxyethyl)phosphine; F2FlAsH: 4′,5′-bis(1,2,3-dithioarsolan-2-yl)-2′,7′-difluorofluorescein; FlAsH: 4′,5′-bis(1,3,2-dithioarsolan-2-yl)fluorescein; βγM: Dimer of β1 and StrepII and CCKACC labeled γ2-subunits.

## Competing interests

The authors declare that there are no competing interests.

## Authors’ contributions

LT expressed and purified Gi proteins, carried out spectrophotometric and fluorescence anisotropy measurements and analyzed and interpreted most of the data and drafted the manuscript. SK carried out fluorescence lifetime measurements, interpreted the data and contributed to overall experimental design, conception and interpretation of the results. AR contributed to overall experimental design, conception and interpretation of results and gave final approval for publication of the results. All authors read and approved the final manuscript.

## Supplementary Material

Additional file 1: Figure S1 Fluorescence emission and excitation spectra of F_2_FlAsH (solid line), F_2_FlAsH+ αolf (dotted line) or F_2_FlAsH+αolf+GTPγS (dashed line). Emission was recorded at 550 nm and excitation at 480 nm. Data are presented as mean ±SD. Click here for file

Additional file 2: Figure S2 Fluorescence lifetime decay curves in the frequency domain of fluorescein (A), F_2_FlAsH (B) F_2_FlAsH+GTPγS (C), F_2_FlAsH+CCPGCC-motif containing peptide (D), F_2_FlAsH+α13 (E), F_2_FlAsH+α13+GTPγS (F), F_2_FlAsH+βγM (G), F_2_FlAsH+αolf (H) and F_2_FlAsH+αolf+GTPγS (I). Fluorescence lifetimes and corresponding fractions were calculated by global fitting of phase shifts (blue) and demodulations (red) that were beforehand computed from measured data as a function of 11 frequencies. Curves are presented as 1-component (dotted line), 2-component (solid line) or 3-component (dashed line, in case I) fits. Click here for file
