# Supplementary figures and images for: Biarsenical ligands bind to endogenous G-protein α-subunits and enable allosteric sensing of nucleotide binding
Source: BMC Biochem. 2013 Dec 17;14:37. doi: 10.1186/1471-2091-14-37 (PMC3878488; doi:10.1186/1471-2091-14-37)

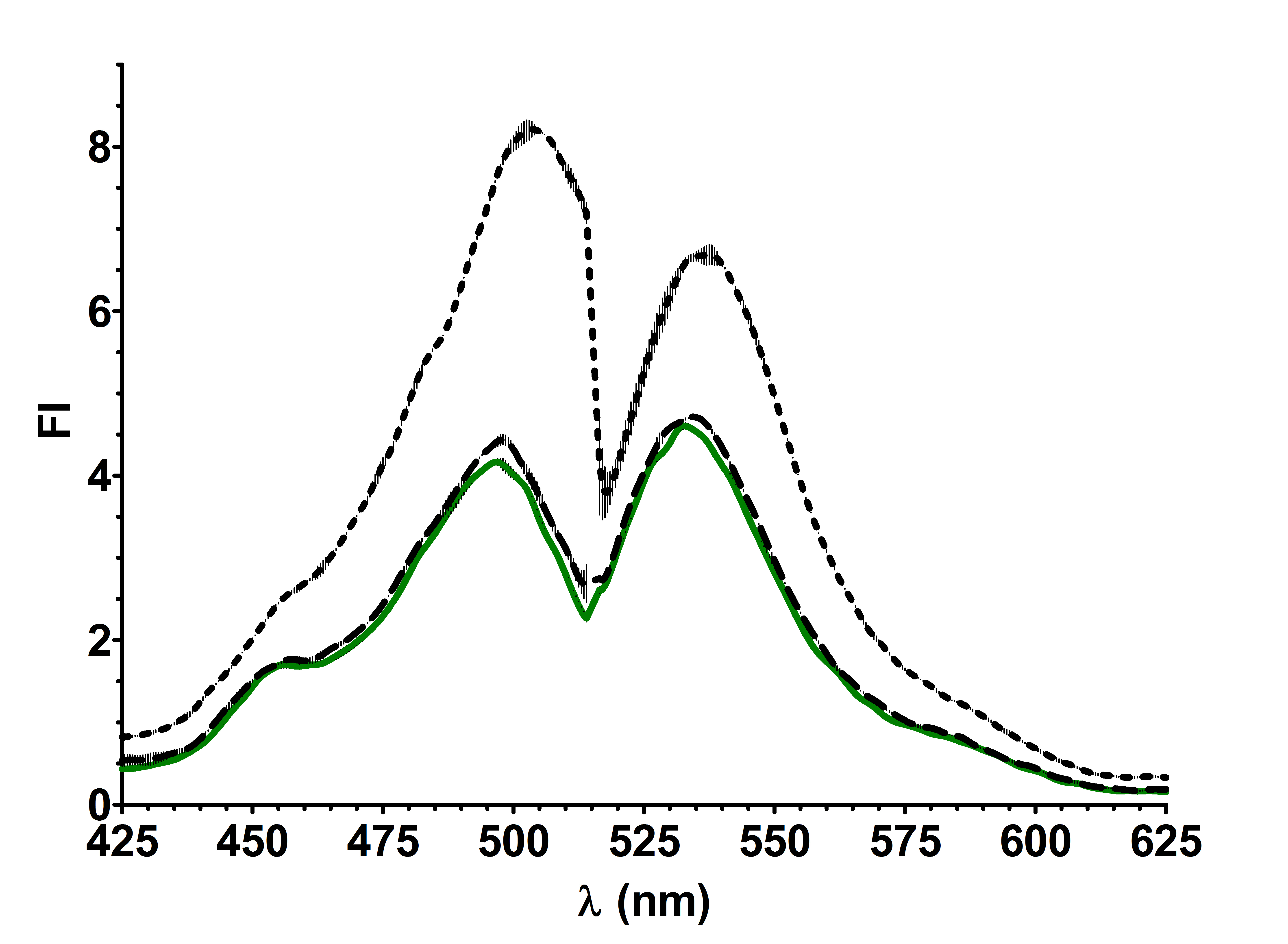

Supplement: Additional file 1: Figure S1 — Fluorescence emission and excitation spectra of F2FlAsH (solid line), F2FlAsH+ αolf (dotted line) or F2FlAsH+αolf+GTPγS (dashed line). Emission was recorded at 550 nm and excitation at 480 nm. Data are presented as mean ±SD. [file 1471-2091-14-37-S1.jpeg]

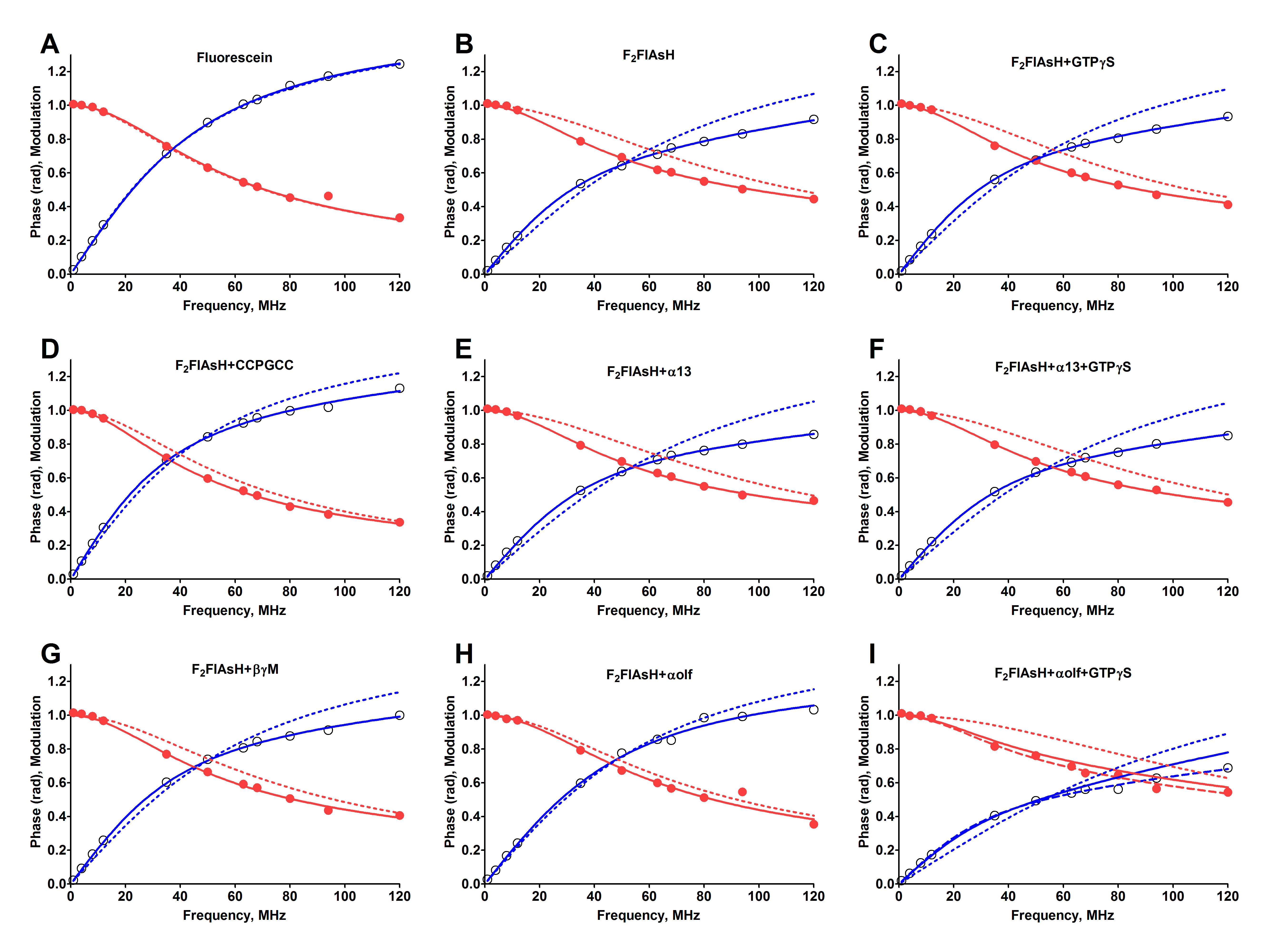

Supplement: Additional file 2: Figure S2 — Fluorescence lifetime decay curves in the frequency domain of fluorescein (A), F2FlAsH (B) F2FlAsH+GTPγS (C), F2FlAsH+CCPGCC-motif containing peptide (D), F2FlAsH+α13 (E), F2FlAsH+α13+GTPγS (F), F2FlAsH+βγM (G), F2FlAsH+αolf (H) and F2FlAsH+αolf+GTPγS (I). Fluorescence lifetimes and corresponding fractions were calculated by global fitting of phase shifts (blue) and demodulations (red) that were beforehand computed from measured data as a function of 11 frequencies. Curves are presented as 1-component (dotted line), 2-component (solid line) or 3-component (dashed line, in case I) fits. [file 1471-2091-14-37-S2.jpeg]
